# Ultrastructural localisation of protein interactions using conditionally stable nanobodies

**DOI:** 10.1371/journal.pbio.2005473

**Published:** 2018-04-05

**Authors:** Nicholas Ariotti, James Rae, Nichole Giles, Nick Martel, Emma Sierecki, Yann Gambin, Thomas E. Hall, Robert G. Parton

**Affiliations:** 1 The University of Queensland, Institute for Molecular Bioscience, Queensland, Australia; 2 EMBL Australia Node in Single Molecule Sciences, School of Medical Science, The University of New South Wales, Sydney, New South Wales, Australia; 3 The University of Queensland, Centre for Microscopy and Microanalysis, Brisbane, Queensland, Australia; UT Southwestern Medical Center, United States of America

## Abstract

We describe the development and application of a suite of modular tools for high-resolution detection of proteins and intracellular protein complexes by electron microscopy (EM). Conditionally stable GFP- and mCherry-binding nanobodies (termed csGBP and csChBP, respectively) are characterized using a cell-free expression and analysis system and subsequently fused to an ascorbate peroxidase (APEX) enzyme. Expression of these cassettes alongside fluorescently labelled proteins results in recruitment and stabilisation of APEX, whereas unbound APEX nanobodies are efficiently degraded by the proteasome. This greatly simplifies correlative analyses, enables detection of less-abundant proteins, and eliminates the need to balance expression levels between fluorescently labelled and APEX nanobody proteins. Furthermore, we demonstrate the application of this system to bimolecular complementation (‘EM split-fluorescent protein’), for localisation of protein–protein interactions at the ultrastructural level.

## Author summary

The use of enzymatic tags such as the ascorbate peroxidase (APEX) for electron microscopic detection of proteins is changing electron microscopy (EM) in the same way that the use of GFP and related proteins caused a revolution in light microscopy. We previously developed expression plasmids encoding GFP-binding peptide (or nanobody) fused to APEX, which allows EM localisation of GFP-tagged proteins in vivo. Here, we have generated conditionally stable GFP- and mCherry-binding nanobodies fused to APEX. Using co-transfection of these APEX nanobodies with fluorescent-tagged constructs, we recruit APEX and detect the tagged proteins by electron microscopy. As unbound conditionally stable nanobodies are efficiently degraded by the proteasome, the signal to noise ratio is dramatically reduced. This enables detection of less abundant proteins and eliminates the need to balance expression levels between fluorescent-labelled and APEX nanobody constructs. Furthermore, and perhaps most exciting, is our application of this method to bimolecular fluorescence complementation—in which two tagged proteins interact—allowing the detection and localisation of protein-protein interactions in EM.

## Introduction

Rapid and reliable protein localisation is critical for the functional characterisation of any protein of interest (POI). Traditionally, this has been achieved through antibody-mediated methods or tagging with a fluorescent protein, such as GFP. The recent emergence of nanobodies (small, single-domain antibodies amenable to cellular expression) has allowed the development of new biotechnological tools based on the detection of epitopes in living cells [1,2], although the availability of defined variable domains for antigen binding remains limiting. At the same time, the use of enzymatic tags such as the soybean ascorbate peroxidase (APEX) for ultrastructural detection of proteins provides an alternative to the use of traditional antibody labelling in electron microscopy (EM) [3], with the advantage of protein localisation throughout the depth of whole cells or tissues making it compatible with the latest revolutionary 3D EM methods [4].

We have previously generated expression plasmids that encode a GFP-nanobody/binding peptide (GBP) for high-resolution detection of GFP-tagged proteins by electron microscopy. To achieve this, we genetically fused the GBP nanobody to the well-characterized soybean-derived enzyme APEX. When APEX–GBP is expressed in the presence of any GFP-tagged POI, its localisation can be determined by transmission EM following processing [5,6].

Here, we have developed and characterized a new suite of APEX/nanobody-mediated tools. As GFP and mCherry are the most broadly used fluorescent proteins in cell biology, we used cell expression to screen a library of putative mCherry-binding peptides (ChBPs) by single-molecule coincidence detection. We demonstrate the utility of a single mCherry nanobody for high-resolution, EM-based analysis of protein distribution and use this probe for correlative analyses. Furthermore, we generate conditionally stable (cs) nanobodies for both GFP and mCherry fused to APEX and show that degradation of unbound cs nanobodies by the proteasomal system reduces background APEX signals and results in an increased signal-to-noise ratio. Finally, we show that the new suite of APEX nanobody tools opens up entirely new avenues for EM localisation through the application of the csAPEX-nanobody system to bimolecular fluorescence complementation, allowing the detection and localisation of intracellular protein-protein interactions at the ultrastructural level.

## Results and discussion

**Fig 1:**
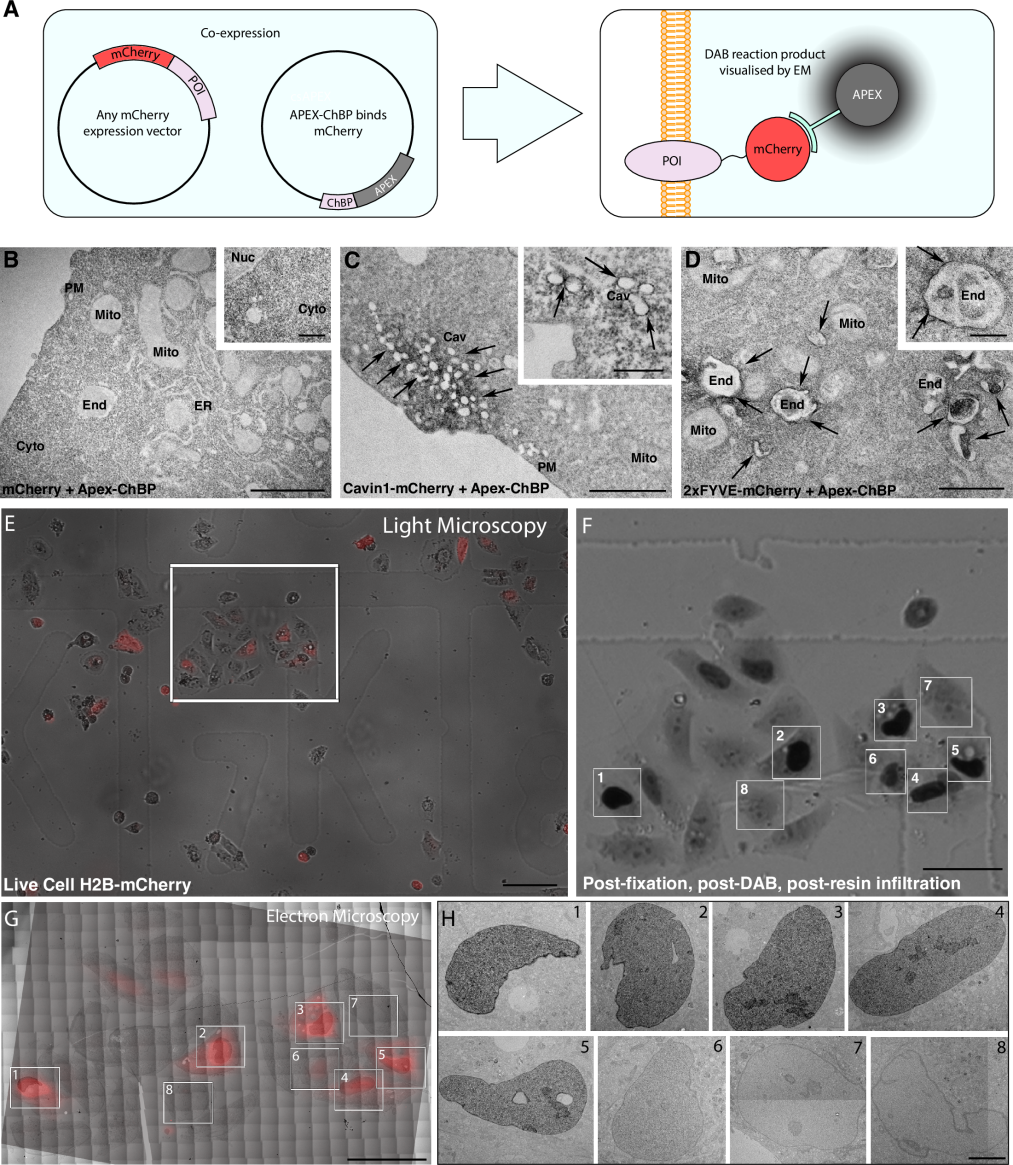
Modular detection of mCherry-tagged proteins using APEX-tagged ChBPs. A) Schematic of cell-based transfection of modular APEX-ChBP and any mCherry-tagged POI. B-D) Electron micrographs of BHK cells co-expressing APEX-ChBP and B) mCherry, C) mCherry-Cavin1, and D) 2xFYVE-mCherry; arrows highlight areas of enriched electron density. Note the increased density in the cytoplasm compared to mitochondria. Scale bars: lower magnification = 1 μm; insets = 500 nm. E-H) CLEM-based detection nls-mCherry–transfected cells using APEX-ChBP. E) 10x magnification of stacked bright field and epifluorescent images of live BHK cells transfected with H2B-mCherry and APEX-ChBP. The grid coordinate (_7_K) can be resolved in the bright field image. White box = region of interest. F) Bright field image of flat-embedded cells after removal of the coverslip and tissue culture dish (corresponds to the region of interest from [E]). Significant DAB reaction product can be resolved in the nucleus of cells transfected with the higher expression of the H2B-mCherry. Eight different cells were selected for higher-resolution EM analysis. G) Montaged electron micrographs of the region of interest correlated with red channel epifluorescence image from (E). H) High-resolution transmission electron micrographs of transfected cells (regions 1 to 8, respectively) demonstrated restricted electron density within the nuclei of high-expressing cells (regions 1 to 5) and low-expressing cells (region 6) and no increased electron density above background in untransfected cells (regions 7 and 8). Scale bars: E = 100 μm, F–G = 50 μm, H = 5 μm. DAB, 3,3′-Diaminobenzidine; APEX, ascorbate peroxidase; BHK, baby hamster kidney; Cav, caveolae; CLEM, correlative light and electron microscopy; ChBP, mCherry-binding peptide; Cyto, cytoplasm; EM, electron microscopy; End, endosome; ER, endoplasmic reticulum; H2B, Histone 2B; Mito, Mitochondria; nls-mCherry, nuclear localized mCherry; Nuc, nucleus; PM, plasma membrane; POI, protein of interest.

To date, no modular systems exist to sensitively detect mCherry-tagged POIs to high-resolution for transmission electron microscopy. Therefore, we initially sought to generate a modular APEX-ChBP expression vector. We screened six sequences previously shown to have affinity for mCherry [7] by fluorescence cross-correlation spectroscopy in *Leishmania tarentolae* cell-free lysate [8]. Each peptide was first expressed fused to the open reading frame of GFP and assayed for self-association or cross-reactivity with GFP (S1A–S1F Fig). ChBP1 and ChBP2 behaved as monomeric proteins (S1A and S1B Fig), whereas ChBP3, ChBP4, ChBP6, and ChBP8 demonstrated bursts of GFP signal above baseline monomeric protein behaviour (S1C–S1F Fig), indicating a propensity for self-association. We next performed single-molecule coincidence detection after co-expression of mCherry-Caveolin1 (Cav1) [9]. mCherry-Cav1 was selected as it generates stable, uniform, and membrane-associated oligomeric Cav1, resulting in highly clustered mCherry tags within the confocal volume. Co-expression of GFP-tagged ChBP1, ChBP3, ChBP4, and ChBP6 with mCherry-Cav1 did not result in significant coincidence between mCherry-Cav1 and GFP-tagged ChBP1, suggesting that these peptides are inefficient at binding the mCherry tag in this context (S1A and S1C–S1E Fig second and third panels). However, ChBP2-GFP and ChBP8-GFP demonstrated a considerable coincidence between the GFP-tagged ChBP and mCherry-Cav1, with a coincidence ratio of Cherry to Cherry and GFP of approximately 0.5, indicating a 1:1 binding ratio of GFP to Cherry (S1B and S1F Fig second and third panel). We selected ChBP2 as the best-performing peptide in our analysis and incorporated this into our modular expression system (Fig 1A; mammalian expression vector hitherto termed APEX-ChBP).

To verify that this construct could be used for high-resolution EM, we co-transfected baby hamster kidney (BHK) cells with APEX-ChBP and three different subcellular markers: (i) mCherry to denote the cytoplasm, (ii) mCherry-Cavin1 to denote caveolae on the plasma membrane (PM), and (iii) 2xFYVE-mCherry to denote early endosomes. Co-expression of the soluble APEX-ChBP and mCherry (with subsequent DAB reaction in the presence of H_2_O_2_ and post-fixation with osmium tetroxide [OsO_4_]) resulted in the accumulation of electron density in the cytoplasm of transfected cells (Fig 1B). This observation closely mirrored the expression of GFP with APEX-GBP [6]. Cavin1 is a critical structural component of plasma membrane microdomains termed ‘caveolae’ and, when present at the PM, resides only within these domains [10]. When APEX-ChBP was co-transfected with mCherry-Cavin1, the electron density generated by the APEX tag and the DAB reaction was restricted to the plasma membrane at structures with morphologies consistent with caveolae (Fig 1C). Finally, we attempted to localize the phosphoinositide (PI) probe 2xFYVE-mCherry (a marker of PI(3)P lipids), which are highly enriched within early endosomes [11]. Co-expression of 2xFYVE-mCherry and APEX-ChBP resulted in the specific accumulation of electron density surrounding structures consistent with early endosomal morphology (Fig 1D). These data demonstrate that our APEX-ChBP vector can be used to localize mCherry-tagged proteins at ultrastructural resolution. As shown in Fig 1E–1H, use of the APEX-ChBP system is compatible with efficient correlative light and EM. Because the APEX2 probe is visible under both light and EM, this represents a simple alternative to more complex and currently widely used CLEM methods.

**Fig 2:**
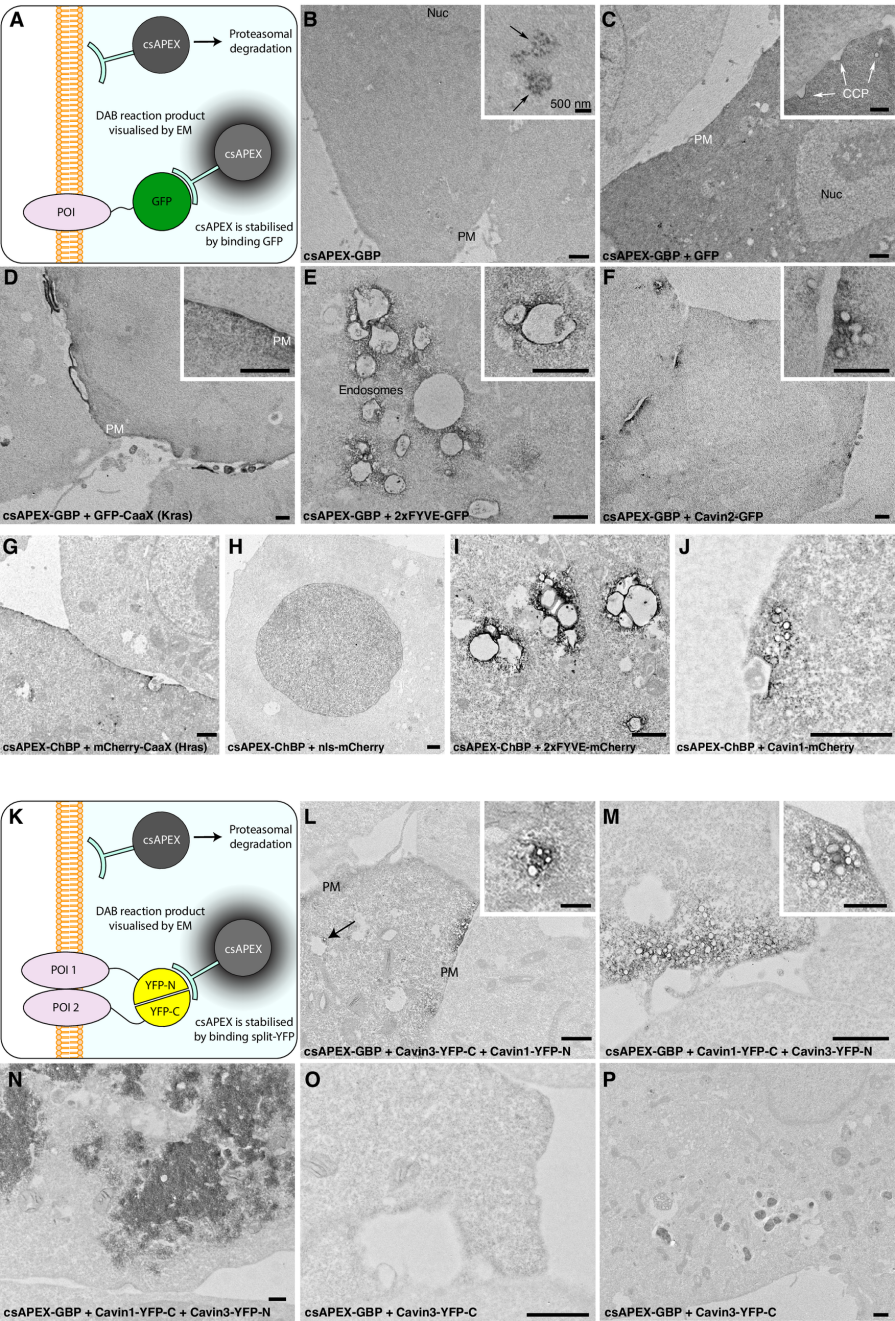
Conditional stabilisation of GBP and ChBP, and detection of protein–protein interactions using bimolecular fluorescence complementation. A) Schematic illustrating detection of GFP-tagged POIs using csAPEX-GBP. The probe is degraded by the proteasome unless stabilized by interactions with a GFP-tagged protein, resulting in loss of any nonspecific, electron-dense APEX signal when csAPEX-GBP does not bind to its target. B) csAPEX-GBP shows minimal signal when expressed in cells lacking GFP-tagged proteins; only a low level of labelling is detectable in specific regions of a subset of cells (inset, arrows). In contrast, cells co-expressing soluble GFP together with csAPEX-GBP show a strong cytosolic signal (C, quantitated in S2 Fig. A). D-F) Examples of subcompartment-specific labelling in cells expressing GFP-tagged POIs associating with the PM, the early endosomes, and caveolae, respectively. G-H) Examples of subcompartment-specific labelling in cells expressing mCherry-tagged POIs associating with the PM, nucleus, early endosomes, and caveolae, respectively. K-P) Co-transfection of BHK cells with constructs tagged with each half of split YFP along with csAPEX-GBP gives strong and specific labelling at sites of protein–protein interactions. K) Schematic illustrating detection of interactions between two POIs tagged with different halves of a split YFP. csAPEX-GBP is able to bind only when the YFP pair is fully reconstituted and folded. In the absence of a correctly folded GFP derivative, csAPEX-GBP is degraded by the proteasome. L) Cavin1-YFP-N and Cavin3-YFP-C co-expression gives specific labelling associated with PM pits and vesicular profiles characteristic of caveolae. Note the specificity of the labelling, which allows identification of Cavin1/Cavin3 complexes associated with both surface caveolae and putative endocytic caveolar carriers associated with intracellular compartments (arrow). Further examples are shown in S2B and S2C Fig. M) Reciprocal experimental conditions with specific fragments of YFP switched between constructs gives consistent labelling. N) Cells with an abnormally high transfection level show intracellular aggregates of Cavin (compare with caveolar labelling in L and M). O) Control cells transfected with just one split GFP half and csAPEX-GBP show no labelling in the majority of cells. P) APEX positive inclusions are seen in a small percentage of control cells. These are clearly distinguishable from the specific staining of the recombined protein complex (L-M). Further examples are shown in S2 Fig. D. Scale bars: lower magnification = 1 μm; insets = 500 nm. BHK, baby hamster kidney; CCP, clathrin-coated pits; ChBP, mCherry-binding peptide; cs, conditionally stable; GBP, GFP-nanobody/binding peptide; PM, plasma membrane; POI, protein of interest.

The modular system for EM detection of fluorescently tagged POIs involves recruitment of APEX-tagged binding peptides to the fluorescent protein (FP). Any unbound APEX nanobody will produce a diffuse cytosolic pool that will hinder detection of the POI and reduce the signal-to-noise ratio, particularly for low-abundance antigens. Recent work using the GBP nanobody has shown that manipulation of specific conserved residues produces a cs protein that is rapidly degraded by the proteasomal system in the unbound state [2]. We used this knowledge to generate csAPEX-GBP; (schematically depicted in Fig 2A) and introduced the analogous residue changes to APEX-ChBP (generating csAPEX-ChBP). Expression of csAPEX-GBP in cells lacking GFP co-expression resulted in only negligible cytosolic APEX signal (Fig 2B); however, in a small number of cells, restricted electron density was observed in a punctate distribution (Fig 2B inset). We hypothesise that this signal represents the residual expression of APEX-GBP in the process of proteasomal degradation. In contrast, co-expression of GFP produced a strong cytosolic signal (Fig 2C, quantitated in S2A Fig) and a complete loss of the punctate distribution observed in the csAPEX-GBP alone. The csAPEX-GBP protein showed efficient recruitment to different cellular compartments, including the plasma membrane, endosomes, and caveolae, showing the functionality of the csAPEX-GBP construct for detection of any GFP-tagged protein (Fig 2D–2F). Consistent results were obtained with csAPEX-ChBP- and mCherry-tagged markers (Fig 2G–2J).

To confirm efficient degradation of our new, conditionally stable csAPEX-ChBP via the proteasomal pathway, we used the well-established proteasome inhibitor MG132 [12]. Cells expressing csAPEX-ChBP alone showed negligible reaction product following the DAB reaction, whereas cells expressing both csAPEX-ChBP and cytoplasmic mCherry showed intense staining throughout (S2B Fig). However, following a 5-h supplementation with 10 μM MG132, cells expressing csAPEX-ChBP alone retained DAB staining in the cytoplasm, indicating that, under normal conditions, csAPEX-ChBP is degraded by the proteasome.

Bimolecular fluorescence complementation (BiFC) is a technique for testing pairwise protein-protein interactions in fixed or living cells by genetically tagging candidates with different halves of a “split” fluorescent protein [13]. If these candidates attain sufficient proximity, the full length fluorescent protein is reconstituted, can fold and emit photons under excitation by a suitable wavelength of light. We hypothesised that by using the conditionally stable APEX nanobody system, we should be able to extend the resolution of bifluorescence complementation to the ultrastructural level. Indeed, the nanobody binding site in GFP (and its variants) straddles the split site in commonly used BiFC pairs [13,14]. Furthermore, folding is absolutely required for the GFP–nanobody interaction, such that recognition of the unfolded halves of the split protein by GBP is a theoretical impossibility. Using this technique, we were able to directly visualize interactions between Cavin1 and Cavin3 by EM using split mVenus, a YFP derivative recognised by the GBP. We transfected BHK cells with vectors encoding Cavin1 fused to the N-terminal fragment of mVenus, (Cavin1-mVenus^1–155^), Cavin3 fused to the C-terminal fragment (Cavin3-mVenus^156–239^), and our csAPEX-GBP construct, schematically represented in Fig 2K. Using this technique, we were able to delineate surface caveolae and putative endocytic caveolar carriers associated with intracellular compartments (Fig 2L, further examples in S2C and S2D Fig). The reciprocal experiment, in which the N- and C-terminal fragments of mVenus were exchanged, showed similar results (Fig 2M). Unusually high-expressing cells were occasionally visible, showing aggregation of intracellular Cavin recognised by csAPEX-GBP (Fig 2N). Transfection with just one-half of the split YFP most commonly showed no cytoplasmic staining (Fig 2O). However, inclusions of increased density were sometimes noted in these controls (Fig 2P, further example in S2E Fig) and were absent from untransfected samples. This staining was clearly distinguishable from the specific signal shown in Fig 2L and 2M, although the importance of such controls is emphasized, particularly since different cell types may contain different numbers of proteasomes. These results clearly demonstrate that protein–protein interactions can be effectively visualized using bimolecular fluorescence complementation at the ultrastructural level using csAPEX-GBP.

In summary, we have utilised cell-free expression and single-molecule analysis to screen a number putative ChBP for association with mCherry-tagged Caveolin-1. The single nanobody we identify is a selective, high-affinity binder of the mCherry tag, lacks detectable self-aggregation or cross-reactivity with GFP, can be linked to APEX for high-resolution analysis of mCherry-tagged proteins in cell culture systems, and is compatible with correlative light and EM. We have also employed conditional stabilisation of both GFP and mCherry binding nanobodies fused to APEX2 which results in the generation of an APEX reaction product only when bound to their target fluorescent proteins. By degrading unbound APEX-BP protein, this modification facilitates an improved signal-to-noise ratio and circumvents any potential oversaturation of the APEX-BP vector. Finally, we have coupled the csAPEX-GBP system with bimolecular fluorescence complementation. This now allows direct visualisation of intracellular protein–protein interactions at the ultrastructural level, far beyond the resolution of light microscopy. This system is immediately applicable (without any new cloning steps) to any system in which the fluorescent split GFP system has been used. Unlike labelling on sections, APEX methods are compatible with 3D EM methods [4] such as focused ion beam-scanning EM, serial blockface-scanning EM, and electron tomography and can be used in whole animal systems [5]. As cellular function depends not on single proteins but on protein–protein interactions, these methods will be a vital complement to dynamic light microscopic methods.

## Materials and methods

### Single-molecule counting and coincidence detection

Single-molecule spectroscopy was performed as previously described [9]. Briefly, samples (20 μl) were loaded into a custom-made silicone 192-well plate adhered to glass coverslips (ProSciTech Australia). Samples were analysed with two lasers (488 nm and 561 nm) using a Zeiss LSM710 microscope with a Conforcor3 module for single-molecule counting and a single 488-nm laser for aggregation analyses. The fluorescence emission was filtered with 505–540-nm band pass filter (GFP) and 580-nm long-pass filter (mCherry). Measurements were taken with photon counts in the approximate range of 750–2,000 which corresponds to a GFP concentration of around 1–2.5 μg/ml. Three replicates were carried out for each construct pair, and consistent results were obtained for each.

### Cell culture

BHK cells were passaged in Dulbecco’s Modified Eagle Medium (Gibco) supplemented with 10% Fetal Bovine Serum and L-Glutamine. Cells were seeded onto 35-mm culture dishes (TPP), transfected with Lipofectamine 3000 as per the manufacturer’s instructions and processed for EM 24 h later. For bimolecular fluorescence complementation experiments, an 8-h incubation in 50 μM cyclohexamide prior to processing was used to reduce background staining.

### EM

EM was performed exactly as described previously [5,6]. Briefly, cells were fixed with 2.5% glutaraldehyde in 0.1-M sodium cacodylate buffer for 1 h at room temperature. Cells were washed with cacodylate buffer to remove the fixative, then washed with DAB in cacodylate buffer for 1 min and subsequently treated with DAB in cacodylate buffer containing H_2_O_2_ for 30 min at room temperature. Cells were post-fixed with 1% OsO_4_ for 2 min to provide contrast. Cells were then washed in water and serially dehydrated in increasing percentages of ethanol before serial infiltration with LX112 resin in a BioWave microwave (Pelco). Resin was polymerised to hardness at 60°C overnight. Ultrathin sections were cut on an ultramicrotome (UC6: Leica) and imaged at 80 kV on a JEOL1011 transmission electron microscope. Sections were not post-stained.

### Correlative light and EM

Cells were grown on 35-mm gridded MatTek dishes (with an in-plane alphanumeric code) and co-transfected with nls-mCherry and APEX-ChBP. Live cell imaging was performed on an EVOS FL epifluorescent microscope (ThermoFisher Scientific) at 10x and 20x magnification. Cells were processed as described above with the following exceptions. Post-polymerisation, the flat-embedded cells were removed from the dish and the region of interest was trimmed using the now-imprinted grid coordinates on the block face. Ultrathin sections were cut, placed on a slot grid, and imaged on a Tecnai 12 transmission electron microscope fitted with a 4K x 4K LC1100 camera (Direct Electron) at 120 kV under the control of SerialEM. Low-magnification (4,400 XMag) montages were acquired at a binning of 1 and stitched together using the Blendmont program in IMOD. Correlation of light and EM images was performed using Photoshop (Adobe Inc.).

### Constructs and cloning

Split mVenus constructs were made by first removing the Fos and Jun inserts from pcs_kmVenus1-155_FosLZ135-171 and pcs_kmVenus156-239_JunLZ253-289 using EcoRV/SpeI. Human Cavin1 and Cavin3 open reading frames were amplified by PCR using the primer tags forward 5′-AGCGGCGGCGGCTCTGATATC-3′ and reverse 5′-ACAAGAAAGCTGGGTACTAGT-3′ and subcloned using infusion (BD). The series of ChBP-GFP expression vectors for *L*. *tarentolae* expression were constructed by PCR subcloning from the original templates [7] into the cell-free gateway cloning vector ‘N-term 8xHis eGFP pCellFree_G03’ [8] (Genbank KJ541667) using the following primer tags: forward 5′-GGGGACAAGTTTGTACAAAAAAGCAGGCTC-3′, reverse 5′-GGGGACCACTTTGTACAAGAAAGCTGGGTT-3′. Previously described vectors used for expression or subcloning were pmCherry-N1 (Clontech PT3974-5), pEGFP-N1 (Clontech PT3027-5), GFP-CaaX(Kras) [15], GFP-2xFYVEhrs [16], mCherry-2xFYVEhrs [17], Cavin1-mCherry [10], Cavin2-GFP and Cavin3-GFP [18], pCSDEST2 [19], pDEST-Tol2-pA2, p5E-CMV/SP6, pME-mCherry-CaaX (Hras) and p3E-pA [20], APEX2-GBP, mKate2-P2A-APEX2-GBP, and pME-APEX2-NS [6]. All other constructs were made using the Multisite Gateway system (Invitrogen). These new vectors have been deposited in the Addgene repository with the following identifiers: APEX2-csGBP (108874), mKate2-P2A-APEX2-csGBP (108875), APEX2-csChBP (108876), EGFP-P2A-APEX2-csChBP (108877), APEX2-ChBP (108878), EGFP-P2A-APEX2-ChBP (108879), H2B-mCherry (108880), nls-mCherry (108881), pME-nls (108882), pME-H2B (108883), p3E-mCherry (108884), pME-mCherry-NS (108885), mCherry-CaaX(Hras) (108886), mVenusN-Cavin1 (108887), mVenusC-Cavin1 (108888), mVenusN-Cavin3 (108889), mVenusC-Cavin3 (108890), p3E-csGBP (108891), p3E-ChBP (108892), p3E-csChBP (108893), p3E-APEX2 (108894), pME-EGFP-P2A-APEX2-NS (108895), and p3E-APEX2-P2A-EGFP (108896).

## Supporting information

S1 Fig APEX-ChBP for EM-based subcellular localisation of Cherry-tagged proteins. Six putative ChBPs were GFP-tagged and co-expressed in cell free *Leishmania* lysate with mCherry tagged Caveolin1. A) ChBP1, B) ChBP2, C) ChBP3, D) ChBP4, E) ChBP6, F) ChBP8. Left-hand panels show GFP intensity through the confocal volume determined by single-molecule counting over time. Middle panels show simultaneous detection of coincidence of ChBP-GFP and mCherry-Cav1 over time. Right-hand panels show plots of the coincidence ratio between red and green channels. Only ChBP2 demonstrated a lack of self-aggregation/cross-reactivity with GFP (B, left panel), equivalent detection of red and green signal intensity over time (B, middle panel), and a 1:1 coincidence ratio of GFP to mCherry (B, right panel). Data underlying all middle panels is available in S1 Data. ChBP, mCherry binding peptide.
(TIF)

S2 Fig Detection of protein-protein interactions using bimolecular fluorescence complementation. A) Quantitation of the effect of GFP presence on stabilisation of the conditionally stable APEX-GBP. Co-transfection of GFP with the csAPEX-GBP construct results in greater than 40% of cells with cytoplasmic density, compared to approximately 5% with transfection of csAPEX-GBP alone. Chi squared, *p* < 0.0001. See also Fig 2C. B) Validation of proteasome-mediated degradation of conditionally stable ChBP. Cells expressing csAPEX-ChBP alone show negligible reaction product following the DAB reaction (top row), whereas cells expressing both csAPEX-ChBP and cytoplasmic mCherry show intense staining throughout (middle row). Follow a 5-hr supplementation with 10 uM MG132, cells expressing csAPEX-ChBP alone retain DAB staining in the cytoplasm indicating that under normal conditions csAPEX-ChBP is degraded by the proteasome (bottom row). C-E) Further examples of Cavin1-YFP-N and Cavin3-YFP-C co-expression giving specific labelling associated with PM pits and vesicular profiles characteristic of caveolae. See also Fig 2L and 2M. E) Further example of APEX positive inclusions are seen in a small percentage of control cells. See also Fig 2P). Scale bars: B = 20 μm, C–E = 1 μm. Data underlying panel A is available in S2 Data. GBP, GFP binding peptide; PM, plasma membrane.
(TIF)

S1 Data Data used to generate the middle panels in S1A–S1F Fig. (XLSX)

S2 Data Data used to generate the middle panels in S2 Fig. (XLSX)

## Abbreviations

ACRF: Australian Cancer Research Foundation
APEX: ascorbate peroxidase
BHK: baby hamster kidney
BiFC: bimolecular fluorescence complementation
Cav1: Caveolin1
ChBP: mCherry-binding peptide
cs: conditionally stable
EM: electron microscopy
FP: fluorescent protein
GBP: GFP-nanobody/binding peptide
IMB: Institute for Molecular Bioscience
PI: phosphoinositide
PM: plasma membrane
POI: protein of interest
