# Supplementary figures and images for: Ultrastructural localisation of protein interactions using conditionally stable nanobodies
Source: PLoS Biol. 2018 Apr 5;16(4):e2005473. doi: 10.1371/journal.pbio.2005473 (PMC5903671; doi:10.1371/journal.pbio.2005473)

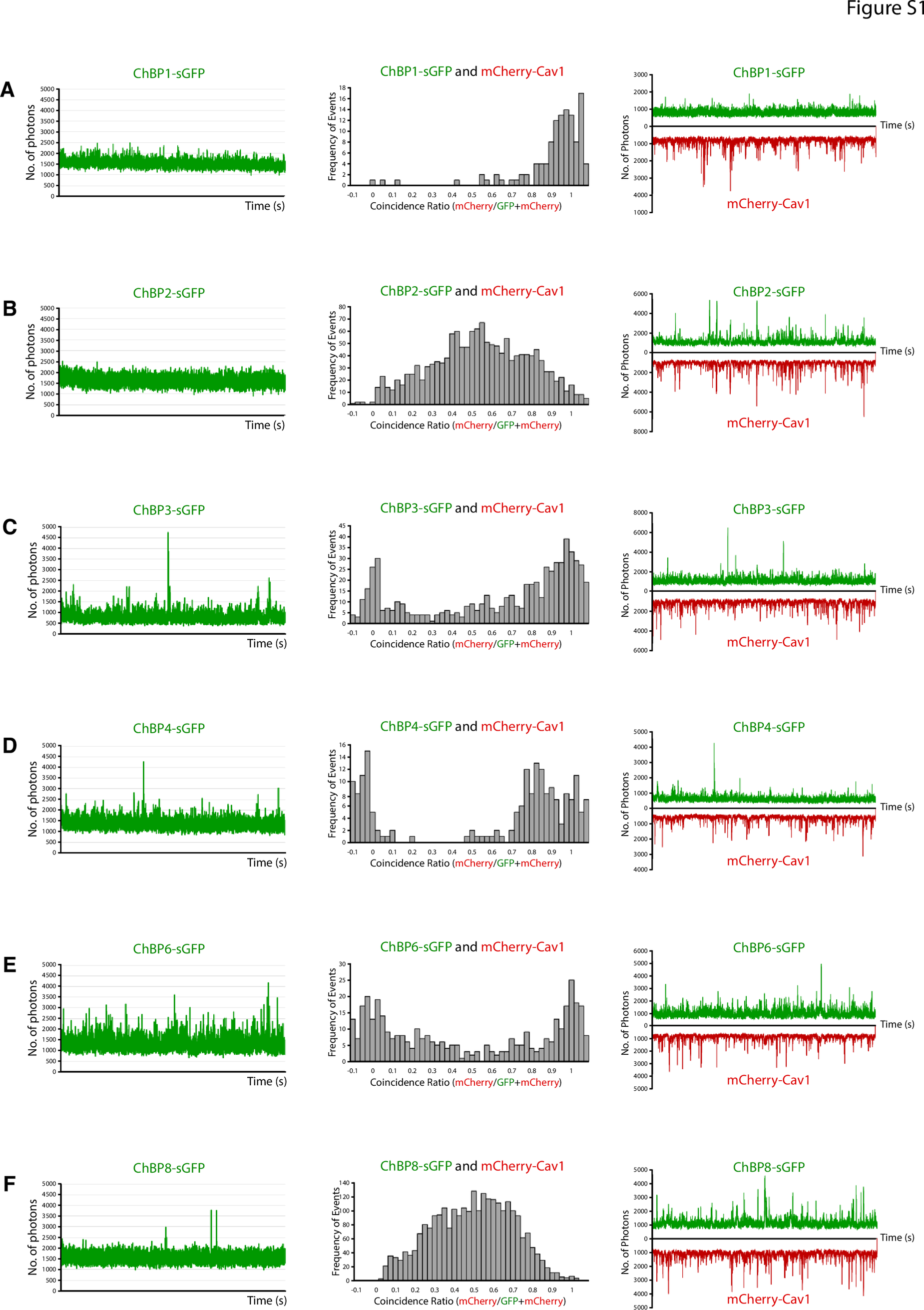

Supplement: S1 Fig — Six putative ChBPs were GFP-tagged and co-expressed in cell free Leishmania lysate with mCherry tagged Caveolin1. A) ChBP1, B) ChBP2, C) ChBP3, D) ChBP4, E) ChBP6, F) ChBP8. Left-hand panels show GFP intensity through the confocal volume determined by single-molecule counting over time. Middle panels show simultaneous detection of coincidence of ChBP-GFP and mCherry-Cav1 over time. Right-hand panels show plots of the coincidence ratio between red and green channels. Only ChBP2 demonstrated a lack of self-aggregation/cross-reactivity with GFP (B, left panel), equivalent detection of red and green signal intensity over time (B, middle panel), and a 1:1 coincidence ratio of GFP to mCherry (B, right panel). Data underlying all middle panels is available in S1 Data. ChBP, mCherry binding peptide. (TIF) [file pbio.2005473.s001.tif]

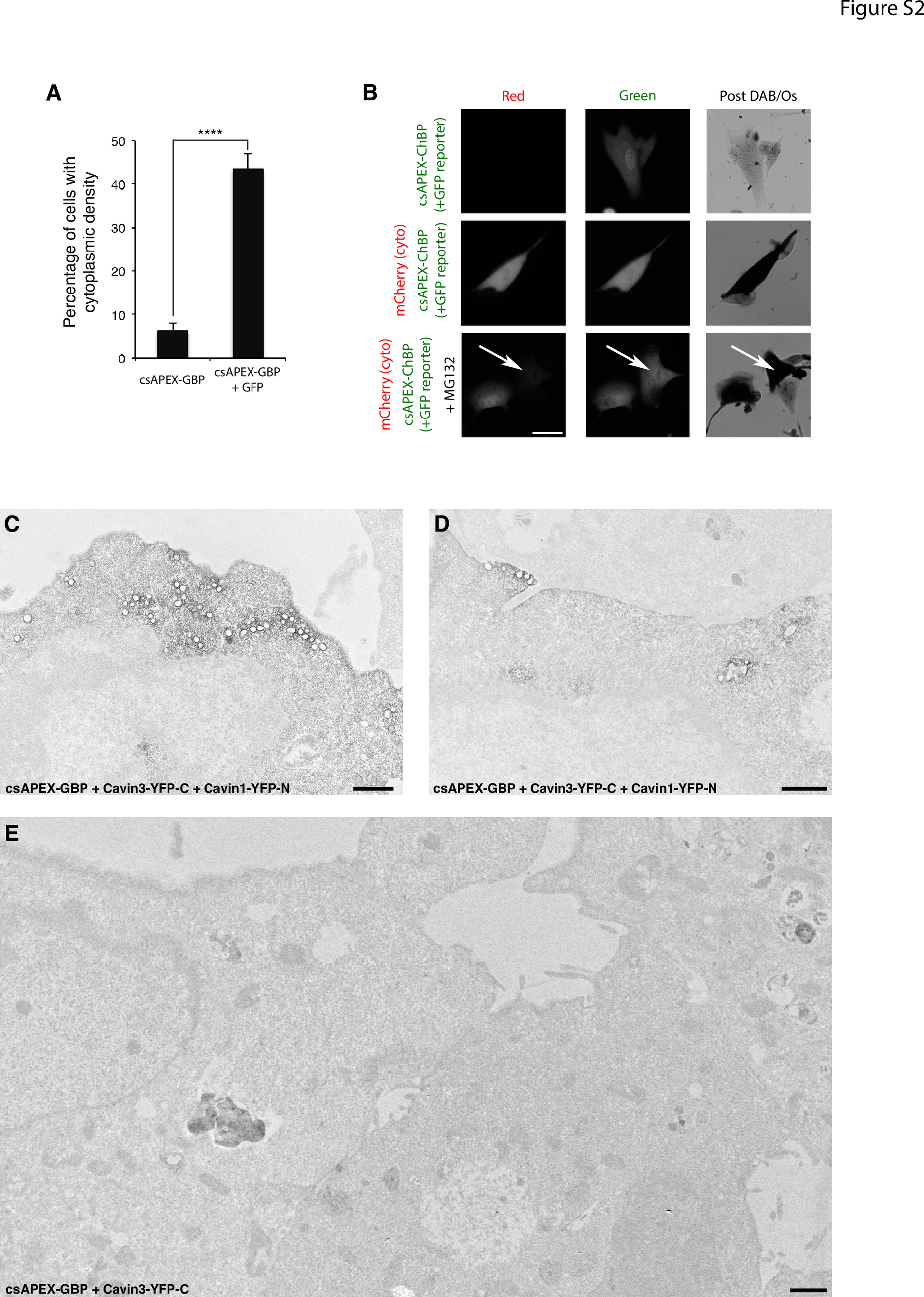

Supplement: S2 Fig — A) Quantitation of the effect of GFP presence on stabilisation of the conditionally stable APEX-GBP. Co-transfection of GFP with the csAPEX-GBP construct results in greater than 40% of cells with cytoplasmic density, compared to approximately 5% with transfection of csAPEX-GBP alone. Chi squared, p < 0.0001. See also Fig 2C. B) Validation of proteasome-mediated degradation of conditionally stable ChBP. Cells expressing csAPEX-ChBP alone show negligible reaction product following the DAB reaction (top row), whereas cells expressing both csAPEX-ChBP and cytoplasmic mCherry show intense staining throughout (middle row). Follow a 5-hr supplementation with 10 uM MG132, cells expressing csAPEX-ChBP alone retain DAB staining in the cytoplasm indicating that under normal conditions csAPEX-ChBP is degraded by the proteasome (bottom row). C-E) Further examples of Cavin1-YFP-N and Cavin3-YFP-C co-expression giving specific labelling associated with PM pits and vesicular profiles characteristic of caveolae. See also Fig 2L and 2M. E) Further example of APEX positive inclusions are seen in a small percentage of control cells. See also Fig 2P). Scale bars: B = 20 μm, C–E = 1 μm. Data underlying panel A is available in S2 Data. GBP, GFP binding peptide; PM, plasma membrane. (TIF) [file pbio.2005473.s002.tif]
